# Characteristics and Drug Utilization of Patients with Hereditary Angioedema in Italy, a Real-World Analysis

**DOI:** 10.3390/healthcare11182509

**Published:** 2023-09-10

**Authors:** Elisa Giacomini, Melania Leogrande, Valentina Perrone, Margherita Andretta, Marcello Bacca, Alessandro Chinellato, Andrea Ciaccia, Mariarosaria Cillo, Renato Lombardi, Daniela Mancini, Romina Pagliaro, Maurizio Pastorello, Cataldo Procacci, Luca Degli Esposti

**Affiliations:** 1 CliCon S.r.l. Società Benefit, Health Economics and Outcomes Research, 40137 Bologna, Italy; melania.leogrande@clicon.it (M.L.); valentina.perrone@clicon.it (V.P.); luca.degliesposti@clicon.it (L.D.E.); 2 Unità Operativa Complessa Assistenza Farmaceutica Territoriale, Azienda ULSS 8 Berica, 36100 Vicenza, Italy; margherita.andretta@aulss8.veneto.it; 3 Programmazione e Controllo di Gestione, ASL Brindisi, 72100 Brindisi, Italy; m.bacca@asl.brindisi.it; 4 Unità Operativa Complessa Farmacia Ospedaliera, Azienda ULSS 3 Serenissima, 30174 Mestre, Italy; alessandro.chinellato@aulss3.veneto.it; 5 Servizio Farmaceutico Territoriale, ASL Foggia, 71121 Foggia, Italy; andrea.ciaccia@aslfg.it (A.C.); lombardi.r@alice.it (R.L.); 6 Dipartimento Farmaceutico, Azienda Sanitaria Locale di Salerno, 84124 Salerno, Italy; mariarosariacillo@gmail.com; 7 Dipartimento Farmaceutico, ASL Brindisi, 72100 Brindisi, Italy; daniela.mancini@asl.brindisi.it; 8 Unità Operativa Complessa Farmaceutica Territoriale, Azienda Sanitaria Locale Roma 5, 00034 Roma, Italy; romina.pagliaro@aslroma5.it; 9 Dipartimento Farmaceutico, Azienda Sanitaria Provinciale di Palermo, 90141 Palermo, Italy; mauriziopastorello@asppalermo.org; 10 Dipartimento Farmaceutico, ASL BAT, 76125 Trani, Italy; aldo.procacci@aslbat.it

**Keywords:** HAE, administrative databases, danazol, long-term prophylaxis

## Abstract

This real-world analysis investigated the characteristics and treatment patterns of patients with hereditary angioedema (HAE) in Italy using the administrative data of health units across Italy. Patients were identified via exemption code or HAE-specific treatments (thus, all known forms, type I, II and, III, were included). The index date was that of first prescription of HAE treatments within the inclusion period (01/2010–06/2021) or of the date of exemption. The number of HAE patients included was 148 (43.2% male, mean age 43.3 years). Gastrointestinal disorders affected 36.5% patients, hypertension affected 28.4%, hypercholesterolemia affected 11.5%, and depression affected 9.5%. The frequent gastrointestinal involvement was further confirmed by the use of antiemetics and systemic antihistamines that doubled after the index date. Among patients enrolled by treatment (*n* = 125), *n* = 105 (84%) were receiving a treatment for acute attacks. This analysis provided insights into the characterization of patients with HAE and their management in Italian clinical practice, suggesting that an unmet therapeutic need could be present for such patients in terms of the clinical burden.

## 1. Introduction

Hereditary Angioedema (HAE), first described in the late 19th century, is a rare genetic disease due to C1 inhibitor (C1-INH) deficiency [1,2], clinically characterized by recurring episodes of skin swelling, abdominal pain, and life-threatening upper airway obstruction due to the involvement of subcutaneous and submucosal areas [3,4].

There are three forms of HAE: type I is the most common, affecting from 80% to 85% of HAE patients and characterized by C1-INH deficiency; type II, found in 15–20% subjects, is characterized by C1-INH dysfunction; and the rare type III, which is typically estrogen-dependent with normal C1-INH activity and levels but brought about due to several gene mutations [2]. 

Diagnostic protocols differ between pediatric and adult subjects. When possible, family screening can allow for early disease identification before symptom onset at pediatric age [5]. In 2017, an international consensus paper provided indications regarding the diagnosis and care of C1-inhibitor-deficient children and adolescents aged under 19 years [6]. In both pediatric and adult patients, a suspected HAE diagnosis initially relies on complement measurement. Given that the disease is transmitted with an autosomal dominant inheritance, the offspring of patients with HAE has a 50% chance of being affected. However, although early HAE identification, ideally prior to symptom onset, might ameliorate the management of the disease, prenatal diagnosis is not commonly used for two main reasons: first, genetic testing is often inconclusive when the mutational status of the parent is unknown; second the measurement of complement levels from umbilical cord blood samples may produce false positives [7]. Disease onset may occur at any age, on average around 10–12 years. Acute attacks, if untreated, commonly worsen within the first 24 h and then resolve in few days [4]. The estimated prevalence ranges between 1/50,000 and 1/100,000 individuals [8]. 

HAE management includes short- and long-term prophylaxis, as well as treatment of acute attacks. The last update released in 2021 of the guidelines by the World Allergy Organization in collaboration with the European Academy of Allergy and Clinical Immunology (WAO/EAACI) [9] recommended short-term prophylaxis, also referred to as situational prophylaxis, to prevent or minimize the consequent risks of acute attacks during exposure situations, such as surgical traumas, dental surgery, or other mechanical interventions that might potentially exacerbate the disease [10]. Preprocedural prophylaxis is based on intravenous (IV) plasma-derived C1-INH concentrate, and attenuated androgens (i.e., danazol). Tranexamic acid has been used in the past but is no longer recommended by guidelines [9]. Moreover, the intent behind long-term prophylactic treatment of HAE is to keep the disease under control via the regular use of therapies to prevent acute attacks [11,12]. The mentioned WAO/EAACI guidelines indicate subcutaneous (subc) or IV plasma-derived C1-INH as preferred frontline long-term prophylaxis. In addition, lanadelumab and berotralstat, two agents targeted against kallikrein (respectively, a monoclonal antibody and an inhibitor), can also be utilized as first-line long-term prophylaxis (respectively, recommendations 16 and 17 of the guidelines) [9]. Androgen derivatives have also been used for first-line long-term prophylaxis in the past; however, currently, the guidelines recommend their use only as second-line long-term prophylactic treatment due to the several side effects [9]. 

Acute HAE attacks should be managed as early as possible with on-demand interventions, and also by means of self-administration or home administration therapy. 

Up to now, poor real-world evidence is available on the patients affected by HAE in Italy. In this context, the present study aimed to analyze the demographic and clinical characteristics of HAE patients of all ages, describing the therapeutic paths and mode of drug utilization in clinical practice in Italy.

## 2. Materials and Methods

### 2.1. Study Design and Data Source 

In Italy, healthcare is provided to all residents in view of the principle of universal healthcare coverage through the Italian National Health Service (INHS) and administered on a regional basis (20 regions). Each region is divided into Local Health Units (LHUs), which are administrative bodies with the function of delivering health services. LHUs manage the administrative databases. Such databases hold information that is meant to be used for administrative purposes in order to track the economic flows from the INHS to the healthcare providers for reimbursement purposes. Administrative databases allow for the identification and description of medicine use profiles in daily clinical practice. Herein, we present a retrospective analysis performed by integrating the administrative databases (demographics, exemption, pharmaceutical, hospitalization, and outpatient specialist services databases) of healthcare entities geographically distributed across north, central, and south Italy, covering a catchment area of 5.4 million health-assisted residents (about 9% of the country population), with data availability spanning from January 2009 up to June 2022. Such databases included all the data concerning health resources reimbursed by the INHS.

In full compliance with the European General Data Protection Regulation (GDPR) (2016/679), privacy was guaranteed by assigning an anonymous univocal numeric code to each subject included in the study. This patient code constituted the electronic linkage between the various databases. All the results are given as aggregated summaries, and are thus never attributable to a single institution, department, doctor, individual, or individual prescribing behaviors. 

### 2.2. Study Population 

From January 2010 to June 2021 (inclusion period), all patients with a diagnosis of HAE were identified by the presence of one or more of the following criteria: (i) at least one active exemption code (a payment waiver code that allow for the avoidance of the economic contribution for services/treatments in the presence of certain diseases) for HAE (code RC0190); (ii) at least one prescription of drugs indicated for the treatment of HAE (anatomical therapeutic chemical (ATC) codes: B06AC01 (C1-inhibitor, plasma-derived) and/or B06AC02 (icatibant) and/or B06AC05 (lanadelumab)); and (iii) at least one prescription of danazol (ATC code: G03XA01) or tranexamic acid oral formulation (ATC code: B02AA02; market authorization code: 021458017, 022019018, 022019020, 044063067), together with at least one hospitalization with the discharge diagnosis ICD-9-CM codes 277.6, 279.8. The time of the first drug prescription or active exemption code for HAE within the inclusion period was considered to be the index date. The characterization period was the time of at least 12 months preceding the index date (all patients had at least 1 year of data availability before the index date). The follow-up comprised all the available period after the index date (all patients had at least 1 year of data availability period after index date).

For the overall HAE population included in the analysis, age at index date, with a distribution organized by age ranges (0–11, 12–17, and ≥18 years), and gender, expressed as the percentage of male subjects, were recorded. During the characterization period, the comorbidity profile was described using the Charlson Comorbidity Index (CCI). Moreover, the most commonly described conditions experienced by HAE patients [4] were also searched in the characterization period, namely: hypertension (at least 1 prescription of antihypertensive drugs with ATC codes: C02, C03; C07-09 or a discharge diagnosis with ICD-9-CM codes: 401–405; or presence of exemption code: A31); hypercholesterolemia (discharge diagnosis with ICD-9-CM codes: 272.0; or at least 1 prescription of lipid modifying agent drugs with ATC code: C10); gastrointestinal disorders (at least 1 prescription of drugs for gastrointestinal disorders with ATC codes: A02-04, A06, A07, A09); and depression (at least 1 prescription of antidepressant drugs with ATC code: N06A). The 10 most frequent medications for clinical conditions other than HAE have been reported at the second level of ATC codes.

In treated patients, the presence of conditions involving different organs/systems were proxied during the characterization period, along with all available follow-up collecting data on the following events: nausea/emesis (at least 1 prescription of antiemetics and antinauseants identified by ATC codes: A04 and A03FA01; furthermore, as a proxy of emesis, proportion of patients with antihistamines for systemic use with ATC R06A have been reported); diarrhea (at least 1 prescription of antidiarrheals by ATC codes: A07D, A07F, A07X); vascular pathologies (at least a discharge diagnosis with ICD-9-CM codes: 444, 452, 453; or for phlebitis and thrombophlebitis with ICD-9-CM code: 451); edema (at least a discharge diagnosis with ICD-9-CM code: 782.3); edema of larynx (at least a discharge diagnosis with ICD-9-CM codes: 478.6, 478.25). 

The most frequent causes of hospitalization during follow-up have also been computed. 

### 2.3. Treatment Patterns for HAE

For all the included HAE patients, the data on prescriptions for acute attacks are as follows: IV plasma-derived C1-inhibitors (ATC code: B06AC01); icatibant (ATC code: B06AC02,); and for prophylaxis, lanadelumab (ATC code: B06AC05). Oral tranexamic acid and oral danazol (ATC code: G03XA01) were collected on the index date and during the available follow-up period. Subcutaneous C1-inhibitors could not be included in the analysis because they have been approved for reimbursement in Italy for the treatment of acute attacks in September 2022, i.e., after the end of the inclusion period of this analysis [13]. Since IV plasma-derived C1-inhibitors can be used both as acute and prophylaxis treatments, when a continuous use was detected, the drug was considered to be dispensed in the prophylaxis setting. 

Treatment patterns and sequences were evaluated in all of the HAE-treated population. Any switch/add-on to a treatment for acute attacks or to a prophylaxis during the follow-up period was recorded. For patients with treatments acute attack at the index date, any switch to a prophylaxis during the follow-up period and any prescription of a prophylaxis drug during the characterization period were considered. For patients under prophylaxis therapy at the index date, any switch/add-on to a prophylaxis during the follow-up period and any prescription of a different prophylaxis drug versus the index date during the characterization period were collected. 

For patients under treatment, the concomitant presence of at least one prescription for analgesics (ATC: N02) or antihistamines (ATC: R06) or corticosteroids (ATC: H02) around 30 days of the index date was also assessed. 

### 2.4. Statistical Analysis

Descriptive statistics are presented as mean ± standard deviation (SD) for continuous variables and as numbers and percentages for categorical variables. In all analyses, the unit was the patient. Following the “Opinion 05/2014 on Anonymization Techniques” drafted by the “European Commission Article 29 Working Party”, the results involving less than 3 patients were not shown since they are potentially traceable to single individuals (thus, they are referred to as NR: not reported). STATA SE version 17.0 SE (StataCorp LLC, College Station, TX, USA) was used for data analysis.

## 3. Results

### 3.1. Characteristics of the Study Population

The HAE population consisted of 148 patients, 64 (43.2%) of which were male. The age at inclusion was 43.3 ± 21.0 years, with 12 (8.1%) of them being aged 0–11 years, 12 (8.1%) between 12 and 17 years, and 124 (83.8%) were adult patients. Mean ± SD follow-up was 5.5 ± 2.4 years.

The comorbidity profiles of the overall HAE patients and those stratified by age classes were described during the characterization period (12 months before the index date) according to frequency of patients with CCI 0, 1 and ≥2, and 10 of the most prescribed medications indicated for concomitant diseases other than HAE (Table 1A). Overall, 58% of the patients fell into the category with CCI 0, around 57% of which being adults and adolescents, but with a peak of 91.7% in pediatric patients (0–11 years). Among the conditions commonly associated with HAE [4], gastrointestinal disorders accounted for 36.5%, followed by hypertension (28.4%), hypercholesterolemia (11.5%), and depression (9.5%). Concerning the most frequent medications, prescriptions for antibacterials were found in 50.0% of patients, followed by drugs for acid-related disorders (33.1%) and systemic corticosteroids (23.0%). 

The overall HAE population was then compared for the same clinical variables with a control group of subjects without HAE of equal numerosity (*n* = 148) and matched for age, gender distribution, and year of inclusion (Table 1B). HAE patients were significantly less represented in the category, with no comorbidities according to Charlson index evaluation (CCI = 0). They experienced more frequent gastrointestinal disorders (36.5% vs. 22.3%, *p* = 0.007) and were more commonly prescribed drugs for acid-related disorders (33.1% vs. 18.2%, *p* = 0.003), systemic corticosteroids (23.0% vs. 11.5%, *p* = 0.009), and beta blockers (15.5% vs. 8.1%, *p* = 0.048). 

During follow-up, the most common causes of hospitalization were those related to the musculoskeletal system and connective tissue and the circulatory system (8.1%), followed by ear, nose, mouth, and throat (4.1%) (Supplementary Table S1). Overall, 125 patients were treated at the index date. As reported in Table 2, an increasing trend in the rate of concomitant conditions commonly found in HAE patients [4] was observed from characterization to follow-up period, which was particularly evident also in the use of systemic antihistamines as antiemetics, which doubled from 10% during the characterization period to 22% during follow-up.

### 3.2. Treatment Patterns and Drug Utilization for HAE

The treatment patterns at the index date in overall HAE patients and those stratified by age are described in Table 3. At the index date, 84% of patients were receiving treatment for acute attacks (56% with IV plasma-derived C1-inhibitor; 28% with Icatibant), while the remaining 16% of patients were under prophylaxis at the inclusion. Concerning the concomitant therapies at the index date (by considering an interval of ±30 days around the index date), in 15 patients of the 105 treated for acute attacks, and in less than 4 of those under prophylaxis, analgesics or antihistamines or corticosteroids were co-administered.

At the index date, 105 patients were under treatment for acute attacks. A prescription of a drug other than the index treatment was given to 47 patients (44.8%) during the follow-up period (specifically, 36 add-on and 11 switch), to 33 (31.4%) during the characterization, and to 26 (24.8%) during both characterization and follow-up period. 

Among the 20 patients who were under prophylaxis at the index date, a prescription of a different drug, i.e., one other than the index treatment, was found in six patients (30.0%), of which four received add-ons during the follow-up, four (20.0%) during the characterization period, and less than four during both the characterization and follow-up periods (not shown for data privacy). The detailed description of index drugs and those prescribed before and after the index date in both acute treatment and prophylaxis are provided in Table 4.

## 4. Discussion

The present analysis characterized a population of HAE patients of all ages, describing the therapeutic paths during prophylaxis and acute attacks and focusing on drug utilization and therapeutic management in the real clinical practice.

The study population consisted mostly of adult patients, with a smaller proportion of children and adolescents. Since patients were identified by exemption code or HAE-specific treatments that do not allow us to discriminate between the known HAE forms, we can assume that patients with type I, II, and III were all included [2]. Demographic characteristics were consistent with a retrospective analysis among the Italian population reporting that almost 47% of HAE patients were male, with a median age of 44–45 years [14]. Unsurprisingly, the comorbidity profile was less severe in younger HAE patients, as documented by the higher proportion of subjects under 18 years old in the category with CCI 0. In addition, most of the drugs indicated for other diseases were, in general, more frequently administered in adults, except antibiotics that were distributed similarly across age classes, and drugs for obstructive airway diseases were more commonly prescribed in adolescents. To date, evidence on the rates of comorbidities in HAE patients in Italy has been scanty. A multinational patient survey reported that gastrointestinal disorders were found in 18% of HAE patients, hypercholesterolemia in 14%, and hypertension in 13% [15]. Likewise, a recent population-based cohort study in Sweden reported an increased risk of cardiovascular disease, dyslipidemia, and autoimmune disease among HAE patients, and data from the national prescription register showed a larger number of prescriptions for hypertension, hypothyroidism, and hyperlipidemia [16]. 

Considering that most of the divergences with our data might be related to the different populations and geographical areas, we find some similar findings, with a consistent proportion of patients reporting gastrointestinal disorders, hypertension, and hypercholesterolemia. Furthermore, treated patients displayed a high clinical burden, suggesting an unmet need for these patients. At present, our data prevent us from identifying with certainty the underlying causes for such complications: however, the comparison with a control group of subjects without HAE of a similar age/gender distribution and year of inclusion revealed that HAE patients were more prone to experiencing gastrointestinal disorders and more frequently prescribed with drugs such as anti-acids, systemic corticosteroids, and beta blockers. Taken together, these results might suggest that such a complex clinical picture might be due to HAE itself (i.e., in case of abdominal localization of attacks) or to adverse effects of treatments. This point deserves further investigation in future studies. 

In our patient population, there was a surprisingly high percentage (close to 18%) of patients on antihypertensive therapy with RAS blockers that are contraindicated in these patients because it may promote an acute attack [17]. However, we collected data using the second-level ATC code C09, which corresponds to “Agents acting on the renin-angiotensin system” and is inclusive of ACEi (alone or in combinations) and angiotensin II receptor blockers (ARBs). An in-depth evaluation was then made to discriminate between the types of RAS blockers administrated in the treated HAE patients using the third-level ATC codes, and we found that only 8 out of 27 received an ACEi, while all the remaining received sartans alone on in combination. Although ARBs are also not indicated for HAE patients, they are less likely to trigger an acute attack since, differently from ACEIs, they do not inhibit the degradation of bradykinin [18]. 

The analysis of the treatment patterns at the index date revealed that about 85% of patients were receiving treatment for acute attacks. This strikingly lower proportion of patients under prophylaxis at inclusion is feasibly due to the criteria used for selection, which demanded the presence of hospitalization discharge codes for patients identified by prophylaxis treatment. This might have led to an underestimation of the numbers of those under long-term prophylaxis as HAE is also largely based on home therapies [19]. The increased possibility of driving patients towards the self-administration management of both prophylaxis and acute attacks might also be a possible explanation underlying the cases wherein patient switched to subsequent therapies other than the index drug (i.e., from an IV administration route to an oral drug), consistent with previous evidence [20,21]. 

Moreover, although the WAO/EAACI 2021 guidelines do not recommend antifibrinolytics [10], we found some patients receiving tranexamic acid. Such a finding should be viewed in consideration of the long observation period of the present analysis, as data were retrieved from the databases from 2009 (12 months before the start of inclusion) to June 2022 (12 months of follow-up, after the closure of inclusion). Therefore, we cannot fully refer to the guidelines released in 2021 for the therapy regimes administrated in our HAE population. There is some evidence in the previous Italian literature to support the utilization of antifibrinolytic drugs, and in particular tranexamic acid, to prevent and treat attacks due to their known ability to inhibit plasmin formation, thus controlling the bradykinin cascade [22]. Moreover, as mentioned before, the inclusion criterion based on the presence of an active exemption code for HAE allowed us to capture all the HAE types, including the rarer familial forms with normal activity of C1-INH and an unknown genetic defect [23]. As stated in the guidelines [10], even though tranexamic acid is considered to be the first choice for prophylaxis of non-histaminergic idiopathic angioedema, its utilization also covers these inherited disease forms with preserved complement function and unknown pathogenesis [23]. 

Perego et al. reported a mean number of 11 attacks during a 1-year period for inpatients with HAE, which was mainly observed among untreated patients, while long-term prophylaxis was associated with a reduced frequency of attacks [24]. Furthermore, our results showed that long-term prophylaxis was administered to nearly all adults, suggesting, in line with the literature [21], an unmet need for pediatric patients who could have had limited treatment options during the time of analysis.

One-month prior or following the index date, a large utilization of analgesics or antihistamines or corticosteroids was noticed, consistent with previous data, indicating that HAE patients are more susceptible to developing allergies or immune disorders [25,26]. 

The main limitations of this study primarily lie in its retrospective observational design. Moreover, we rely on administrative databases, which are primarily intended for administrative purposes. Alongside the advantage of deriving information on a sample of health-assisted patients from real-life clinical practices, administrative databases suffer the limitation of a lack of clinical data related to disease severity and other potential confounding factors. In this regard, given that the population was identified by means of active exemption codes, drug prescriptions, and hospitalization discharge diagnosis, some untreated and non-hospitalized patients might not have been detected. Moreover, since the data reported all resources reimbursed by the NHS, out of pocket resources, such as analgesics and antihistamines, may not be captured, thus providing an underestimation of the comorbidities related to the disease and of the potential adverse events arising from the continuous use of HAE treatments.

Still, administrative databases, due to the high availability of data with periodic updates, are an increasingly used source for health research purposes. Studies based on real-world data can indeed be an important support in obtaining a better understanding of the clinical course and management of patients, especially in cases of rare diseases, where the low sample sizes are often a substantial issue for clinical trials. 

## 5. Conclusions

Herein, we have provided a thorough description of patients with HAE and their therapeutic management in Italian clinical practice. Results showed a high clinical burden related to HAE, which was also observed among treated patients. Indeed, among the latter, an increasing trend of comorbidities after the start of treatment has been reported, which might be due to clinical manifestations of HAE itself, as well as the possible side-effects of the therapies used at the time of the analysis. Furthermore, within the HAE population analyzed, pediatric patients were mostly treated for acute attacks, and almost none of them was prescribed with long-term prophylaxis. Therefore, our findings could underline an unmet therapeutic need with respect to both adult and pediatric patients that should be taken into account in the development of new therapeutic options.

## Figures and Tables

**Table 1:** Comorbidity profile variables of HAE patients stratified by age and overall (A) and in the control population without HAE (B). Comparisons were made between overall HAE vs. the no-HAE control group with the same numerosity (*n* = 148) and similar age/gender distribution and year of inclusion via the chi-square test. Significances have been indicated as * *p* < 0.05 and ** *p* < 0.01.

	A. HAE				B. No-HAE
Characteristics	0–11 Years (*n* = 12)	12–17 Years (*n* = 12)	≥18 Years (*n* = 124)	Overall HAE (*n* = 148)	Control Group (*n* = 148)
CCI = 0	11 (91.7%)	7 (58.3%)	68 (54.8%)	86 (58.1%)	108 (73.0%) **
CCI = 1	NR	4 (33.3%)	36 (29.0%)	41 (27.7%)	30 (20.3%)
CCI ≥ 2	-	NR	20 (16.1%)	21 (14.2%)	10 (6.8%) *
Hypertension, *n* (%)	-	NR	41 (33.1%)	42 (28.4%)	37 (25.0%)
Hypercholesterolemia, *n* (%)	-	-	17 (13.7%)	17 (11.5%)	11 (7.4%)
Gastrointestinal disorders, *n* (%)	NR -	NR	53 (42.7%)	54 (36.5%)	33 (22.3%) **
Depression, *n* (%)	-	NR	13 (10.5%)	14 (9.5%)	7 (4.7%)
Ten most frequent drug prescriptions					
J01-Antibacterials for systemic use	5 (41.7%)	5 (41.7%)	64 (51.6%)	74 (50.0%)	68 (45.9%)
A02-Drugs for acid related disorders	-	NR	48 (38.7%)	49 (33.1%)	27 (18.2%) **
H02-Corticosteroids for systemic use	NR	NR	31 (25.0%)	34 (23.0%)	17 (11.5%) **
M01-Antiinflammatory and antirheumatic products	-	NR	28 (22.6%)	31 (20.9%)	24 (16.2%)
R03-Drugs for obstructive airway diseases	NR	NR	24 (19.4%)	28 (18.9%)	15 (10.1%) *
C09-Agents acting on the renin-angiotensin system	-	NR	26 (21.0%)	27 (18.2%)	29 (19.6%)
C07-Beta blocking agents	-	NR	22 (17.7%)	23 (15.5%)	12 (8.1%) *
B01-Antithrombotic agents	-	NR	20 (16.1%)	21 (14.2%)	13 (8.8%)
C10-Lipid modifying agents	NR -	NR-	17 (13.7%)	17 (11.5%)	11 (7.4%)
C03-Diuretics	-	NR-	17 (13.7%)	17 (11.5%)	8 (5.4%)

Abbreviations: CCI, Charlson comorbidity index; NR, not reported for data privacy (<3 patients).

**Table 2:** Pattern of concomitant conditions (expressed as number of patients with percentage in brackets) in treated HAE patients during characterization and follow-up periods.

	Overall HAE Treated Patients (*n* = 125)	
	Characterization Period	All Available Follow-Up
Hypertension, *n* (%)	33 (26.4%)	47 (37.6%)
Hypercholesterolemia, *n* (%)	14 (11.2%)	22 (17.6%)
Gastrointestinal disorders, *n* (%)	44 (35.2%)	62 (49.6%)
Antiemetics/antinauseants, *n* (%)	-	6 (4.8%)
Systemic antihistamines *, *n* (%)	12 (9.6%)	27 (21.6%)
Depression, *n* (%)	11 (8.8%)	20 (16.0%)

* Proxy of antiemetics. No record was detected for vascular pathologies, edema, and antidiarrheals.

**Table 3:** Treatment patterns in overall HAE patients and those stratified by age classes. Frequencies are expressed as absolute numbers with percentages in brackets.

	HAE Treated			
	Overall (*n* = 125)	0–11 Years (*n* = 8)	12–17 Years (*n* = 12)	≥18 Years (*n* = 105)
**Treatments for acute attacks**	105 (84.0%)	7 (87.5%)	12 (100%)	86 (81.9%)
IV plasma-derived C1-inhibitor	70 (56.0%)	7 (87.5%)	12 (100.0%)	51 (48.6%)
Icatibant	35 (28.0%)	-	-	35 (33.3%)
**Treatment for prophylaxis**	20 (16.0%)	-	-	19 (18.1%)
Tranexamic acid (oral)	NR	-	-	NR
Danazol	4 (3.2%)	-	-	4 (3.8%)
IV plasma-derived C1-inhibitor	13 (10.4%)	NR	-	12 (11.4%)

Abbreviations: IV, intravenous; NR, not reported for data privacy (<3 patients).

**Table 4:** Previous treatments, treatments at the index date, and subsequent treatments in acute and prophylaxis settings.

Patients with Acute Treatment at the Index Date (*n* = 105)			
Previous Treatment	Index Treatment	Subsequent Treatment	*n* (%)
-	IV C1-inhibitor, plasma-derived	-	35 (33.3%)
-	Icatibant	-	16 (15.2%)
Danazol	IV C1-inhibitor, plasma-derived *(all add-on)*	Danazol *(9 add-on)*	11 (10.5%)
-	IV C1-inhibitor, plasma-derived	Plasma-derived C1-inhibitor (*5 switch)*	6 (5.7%)
Other	Other	Other	38 (36.2%)
**Patients with prophylaxis at the index date (*n* = 20)**			
-	Plasma-derived C1-inhibitor	-	9 (45.0%)
Other	Other	Other	11 (55.0%)

## Data Availability

All data used for the current study are available upon reasonable request to CliCon S.r.l., which is the body entitled to data treatment and analysis by participating Local Health units.

## Supplementary Materials

The following supporting information can be downloaded at https://www.mdpi.com/article/10.3390/healthcare11182509/s1: Supplementary Table S1: List of the most frequent hospitalizations among HAE patients during the follow-up period.
