# Supplementary material for: Characteristics and Drug Utilization of Patients with Hereditary Angioedema in Italy, a Real-World Analysis
Source: Healthcare (Basel). 2023 Sep 10;11(18):2509. doi: 10.3390/healthcare11182509 (PMC10530529; doi:10.3390/healthcare11182509)
Supplement: Supplementary file 1 [file healthcare-11-02509-s001.zip › healthcare-2475161-supplementary.pdf]

Supplementary Table S1. List of the most frequent hospitalizations among HAE patients during the follow-up period.

	Overall Pts (N=148)
**DESCRIPTION**	
Musculoskeletal System And Connective Tissue	12 (8.1)
Circulatory System	12 (8.1)
Respiratory System	6 (4.1)
Ear, Nose, Mouth And Throat	6 (4.1)
Pregnancy, Childbirth And Puerperium	6 (4.1)
Skin, Subcutaneous Tissue And Breast	6 (4.1)
Factors Influencing Health Status	5 (3.4)
Hepatobiliary System And Pancreas	5 (3.4)
Myeloproliferative DDs (Poorly Differentiated Neoplasms)	5 (3.4)
Digestive System	5 (3.4)
